# *Lactococcus lactis* As a Versatile Vehicle for Tolerogenic Immunotherapy

**DOI:** 10.3389/fimmu.2017.01961

**Published:** 2018-01-17

**Authors:** Dana P. Cook, Conny Gysemans, Chantal Mathieu

**Affiliations:** ^1^Laboratory of Clinical and Experimental Endocrinology (CEE), KU Leuven, Leuven, Belgium

**Keywords:** *Lactococcus lactis*, mucosal tolerance, immunotherapy, autoimmunity, type 1 diabetes

## Abstract

Genetically modified *Lactococcus lactis* bacteria have been engineered as a tool to deliver bioactive proteins to mucosal tissues as a means to exert both local and systemic effects. They have an excellent safety profile, the result of years of human consumption in the food industry, as well as a lack of toxicity and immunogenicity. Also, containment strategies have been developed to promote further application as clinical protein-based therapeutics. Here, we review technological advancements made to enhanced the potential of *L. lactis* as live biofactories and discuss some examples of tolerogenic immunotherapies mediated by mucosal drug delivery *via L. lactis*. Additionally, we highlight their use to induce mucosal tolerance by targeted autoantigen delivery to the intestine as an approach to reverse autoimmune type 1 diabetes.

## Introduction

The mucosal immune system in close interaction with symbiotic bacteria is constantly working to maintain a homeostatic balance between immune activation, which is necessary against vast amounts of exogenous antigens and noxious stimuli, and immune tolerance toward harmless inhaled or ingested antigens and the host microbiota. Disruption of the mucosal homeostasis can result in inflammatory bowel diseases (IBDs) represented by ulcerative colitis (UC) and Crohn’s disease (CD). Mucosal tolerance, the ability of mucosally administered antigen(s) to regulate local and systemic immune responses has led to new therapeutic approaches to prevent or treat allergies and autoimmune diseases, such as multiple sclerosis (MS), arthritis, uveitis and type 1 diabetes (T1D). In this regard, mucosal (i.e., nasal or oral) drug delivery is generally the preferred treatment route, as it is easy and provides more patient comfort. Moreover, targeting therapeutics to the mucosal surfaces may also display superior efficacy over classic systemic immunotherapies. In this review, the biotechnological potential of genetically modified (GM) *Lactococcus lactis* bacteria for mucosal delivery of biotherapeutics as a means to restore local inflammation and (re-)introduce antigen-specific systemic tolerance will be discussed. The abundant successful preclinical applications of these recombinant *L. lactis* harbor great therapeutic potential and will be covered in detail. In addition, we will provide an overview of therapies using GM *L. lactis* that have been tested in clinical trials and discuss how they can be improved.

## Exploiting Mucosal Tolerance Mechanisms

Mucosal tolerance is the active process involving inhibition of antigen-specific immune responses introduced to the organism *via* the mucosal surfaces as found in the lungs and gastrointestinal tract (GIT). It results in suppression of immunological responses to innocuous antigens and avoids unwarranted pro-inflammatory immune responses (1). In healthy individuals, the gut-associated lymphoid tissue (GALT) will only mount an inflammatory response to danger signals, such as toll-like receptor (TLR) activation, when necessary.

The mechanisms of mucosal tolerance are still not completely elucidated, but it is generally accepted that clonal anergy or deletion of reactive cells and induction of regulatory T cells (Tregs) are the two main effector mechanisms (1, 2). High doses of antigen favor clonal anergy or deletion of reactive cells (3). Anergic T cells form defective immunologic synapses with antigen-presenting cells (APCs) resulting in a hyporesponsive state (4). These cells lose their migratory ability and remain at the site of induction where they display immunosuppressive effects on other T cells in an antigen-independent manner (5). Low-dose oral tolerance favors the induction of Tregs. Mucosal tolerance can be induced in the absence of natural Tregs and is established by *de novo* induction of antigen-specific CD4^+^CD25^+^Foxp3^+^ Tregs in a transforming growth factor (TGF)-β-dependent manner (6). The current view indicates that intestinally induced Tregs (iTregs) result from an interaction with CD103^+^ dendritic cells (DCs). After antigen uptake, these CD103^+^ DCs migrate to the mesenteric lymph nodes where they induce Foxp3^+^ Treg conversion in the presence of retinoic acid, necessary for expression of two gut-homing molecules (CCR9 and integrin α4β7) (7, 8). Gut-homing iTregs return to the lamina propria (LP) where they expand and are instructed by CX3CR1^+^ macrophages to produce IL-10, after which they enter the bloodstream to exert systemic effects (9). Tregs can actively suppress autoreactive T cells in a one-on-one manner; however, they also induce antigen-non-specific immune suppression through “bystander suppression” by secreting anti-inflammatory cytokines (10). Autoreactive T cells that respond to a different antigen than that was mucosally given will therefore also be inhibited. Bystander suppression is useful in diseases with unknown autoantigens, multiple autoantigens, or when there is excess inflammation but no autoantigen (11, 12). It is clear that Tregs are critical for continued immune tolerance in the GIT through active control of innate and adaptive immune responses. Dynamic adaptation of Treg populations to the intestinal tissue microenvironment is key in this process.

Although mucosal tolerance happens throughout the entire lifespan, translating this naturally occurring phenomenon into a therapeutic strategy is not self-evident. Many factors need to be taken into account including antigen choice, dose, route, formulation, timing, and frequency of administration. Inducing therapeutic mucosal tolerance by feeding or inhalation of raw protein is a cumbersome task limited by enzymatic degradation in the GIT or nasal secretions, short half-life due to metabolism, limited bioavailability due to molecular size, loss of tertiary structures or posttranslational modifications (PTMs) necessary for antigen recognition, and finally the high cost of development (13). Bringing protein synthesis to the site of tolerance induction would circumvent these technical obstacles. Many researchers have modified the probiotic *L. lactis* to deliver intact therapeutic bioactive proteins to the GIT. This bacterial strain offers several technical advantages and has been tested in diverse applications.

## *L. Lactis* as Next-Generation Biofactories

### Rational for Choosing *L. lactis*

Non-pathogenic lactic-acid bacteria (LAB), such as particular species of lactococci and lactobacilli, have been handled for centuries in the fermentation and preservation of food. Sequencing of the entire genome of a number of heterofermentative *L. lactis* strains (14, 15) has led to the design of a plethora of genetic tools to engineer these gram-positive bacteria into next-generation mucosal delivery tools for bioactive peptides. Moreover, *L. lactis* strains are specifically important because of their use in the production of probiotic dairy products (16). *L. lactis* consists of three subspecies: *L. lactis* subsp. *cremoris, L. lactis* subsp. *hordniae*, and *L. lactis* subsp *lactis*. The *L. lactis* subsp. *cremoris* MG1363 is the international archetype for LAB genetics; it is a plasmid-free and phage-cured derivative of the dairy starter strain NCDO712-lacking extracellular proteases. The removal of the pLP712 plasmid, which encodes the *lac* operon and proteases necessary for casein degradation, precludes growth in milk thus limiting propagation of this strain outside well-controlled environmental niches (17). Today, there is sufficient knowledge to support the exploitation of GM LAB, and especially the *L. lactis* subsp. *cremoris*, to distribute therapeutic proteins to the mucosal surfaces (18). The widespread historical use of the *L. lactis* strains in the food industry rendered them with an important “generally regarded as safe (GRAS)” status by the Food and Drug Administration (FDA). Moreover, *L. lactis* strains do not colonize the GIT of humans and animals.

### Versatile Protein Delivery Systems

**Figure 1:**
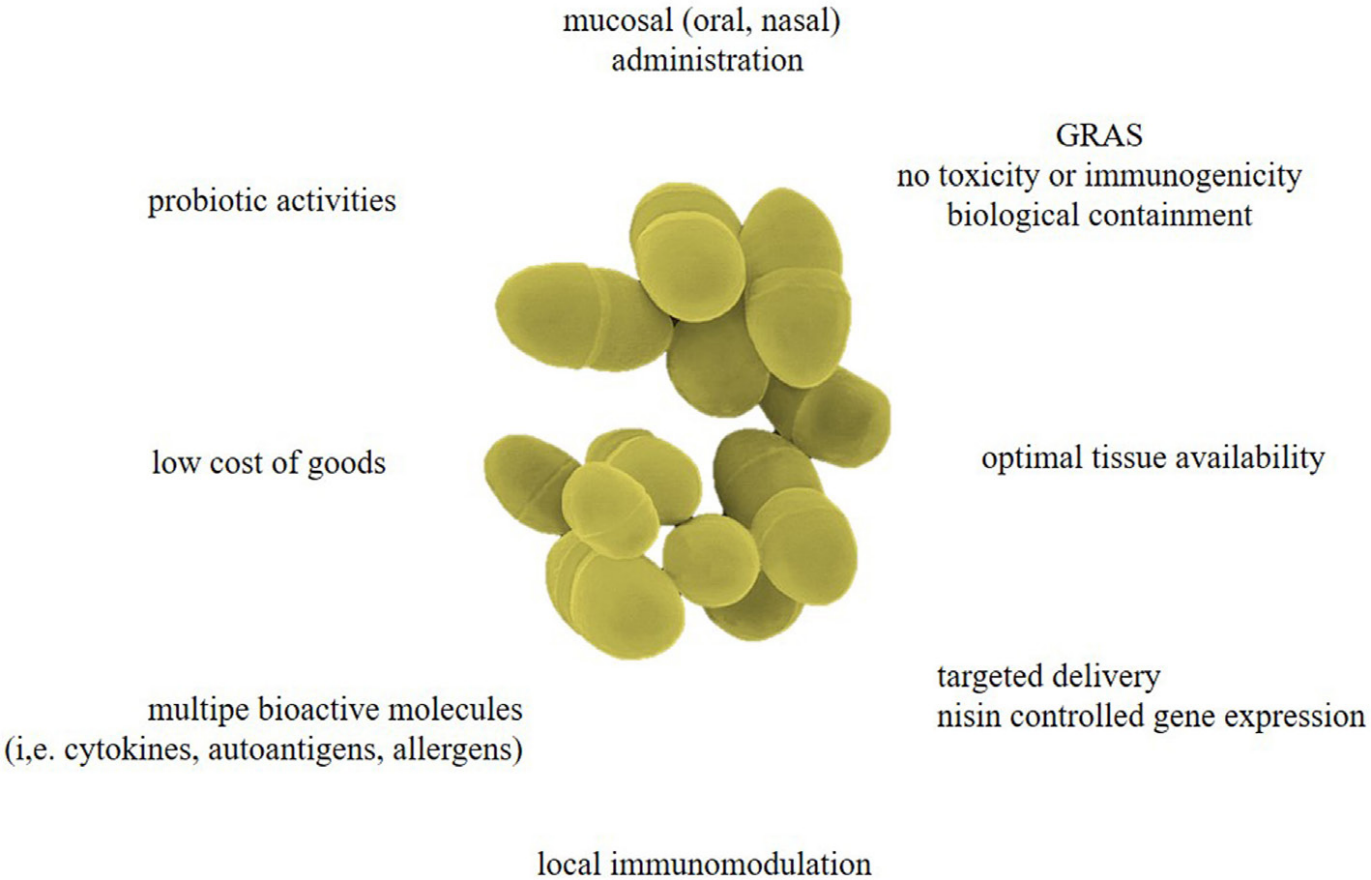
Advantages of *Lactococcus lactis* as designer probiotics for various biopharmaceuticals. As the effectiveness of mucosal-administered antigens diminishes during passage through the gastrointestinal tract (GIT), *L. lactis*-mediated drug delivery could be a safe and low-cost approach for targeted delivery of several bioactive molecules (i.e., cytokines, hormones, antibody fragments, allergens, antigens, etc.), therefore bypassing the adverse effects associated with systemic drug administration. Controlled expression of biopharmaceuticals can offer local immunomodulation and reverse allergy or autoimmunity. These living biofactories have a generally regarded as safe (GRAS) status and can be biologically contained so they cannot colonize the mucosa of the oral cavity or GIT. Moreover, these designer probiotics could reinforce normal immunity to defend the host from infections, inflammatory diseases, and autoimmune responses.

Engineering these bacteria to secrete active therapeutic biologicals can be advantageous for multiple reasons (Figure 1):

(1) The *L. lactis*-based delivery system can circumvent the use of large amounts of crude proteins that for a large part will be broken down by digestive enzymes. Furthermore, soluble proteins have low immunogenicity and stability when given mucosally (i.e., oral, nasal…). The *L. lactis* can survive the entire GIT while expressing one or more bioactive proteins. This is not only cost-effective but eliminates the variation of how much is digested during transit before reaching its target. Since systemic exposure to the therapeutic biological is negligible, the chance of side effects will also be significantly lower. Although reduced viability in the human GIT due to acid sensitivity is an inherent feature of *L. lactis*, this can be limited through proper enteric coating of freeze-dried *L. lactis* (19). Another modification that can offer robust protection against bile-toxicity and gastric-acid assault is intracellular accumulation of trehalose, a known cryoprotectant, by introducing trehalose synthesizing genes (20).
(2) The *L. lactis* strains can be tailored to express heterologous proteins, either constitutive or inducible, depending on the biological need. Strong constitutive promoters for *L. lactis* have been described (21); however, continuous high-level secretion of specific proteins, such as cytokines and interleukins (IL), could induce intracellular accumulation or degradation, potentially leading to cellular toxicity. Therefore, inducible protein expression may be desired when it comes to regulated drug administration. The most successful inducible expression system is the nisin-controlled gene expression (NICE) system where gene expression is proportional to the concentration of the antimicrobial peptide nisin (22). With this flexible system, the level of gene expression can be restrained by the amount of nisin used for the induction and can be upregulated more than 1,000-fold. Other inducible systems also exist, relying on lactose availability, glucose, pH decrease, or zinc starvation (23). As mentioned, inducible gene expression allows control of therapeutic drug delivery; however, additional genetic engineering may hamper clinical use of the *L. lactis* strains. Steidler and colleagues designed the first elegant biological containment system allowing constitutive gene expression as bacterial viability depends on addition of thymidine or thymine, which is not present in the environment and at meagre amounts *in vivo* (24). Briefly, the thymidylate synthase gene *thyA*, which is essential for DNA metabolism, was replaced with a synthetic human gene, in this case human IL-10. This system combines passive containment, as growth is dependent on supplementation of the essential metabolite, i.e., thymidine, with that of active containment, since thymine-less death is bactericidal rather than bacteriostatic as is the case for most other auxotrophies. This system has many advantages including bypassing the use of antibiotic resistance markers which can disseminate to other microbiota.
(3) Secretion of active proteins is often preferred, as this provides the most straight forward interaction with the mucosa. In theory however, the preferred protein can be produced in the cytoplasm, culminate in the cell membrane, or be distributed from the cells to appear in the environment (secreted) or become anchored at the bacterial cellular surface. Different expression vectors such as pCYT, pSEC, and pCWA have been established, to permit protein targeting to be either intracellular, extracellular (secreted form), or cell wall-anchored, respectively (18). When using the pCYT vector, the protein is produced but resides in the bacterial cell in the absence of a signal sequence. As such, this approach protects the protein from degradation but depends on cellular lysis to bring it in the extracellular space. The pCYT and pSEC vectors, in which a nisin-inducible promoter controls expression, should be used in the *L. lactis* NZ9000 strain bearing a *nisR,K* chromosomal cassette, required for nisin signaling. Of interest, *L. lactis* strains have a monolayer cell wall which permits direct extracellular secretion. This method allows immediate contact of the active protein with the mucosa but leaves it more sensitive to gastric digestion and proteolysis. The *L. lactis* strains used for recombinant protein expression only have one extracellular housekeeping protease, high-temperature requirement A (HtrA), keeping the effects of proteolysis to a bare minimum. Secreted proteins need an N-terminal signal peptide (SP) and most often the SP of Usp45, the major extracellular protein of the *L. lactis* bacteria, is used (25). However, this SP does not guarantee efficient secretion and other steps, such as protein trimming, may be required to allow successful expression (26). Protein size, the nature of the SP, and the presence of a pro-peptide are important parameters that may hamper protein secretion. Noteworthy, proteins with molecular mass ranging from <10 to >160 kDa have been efficaciously produced in the *L. lactis* strain. This implies that protein size is not a major problem for heterologous protein production in *L. lactis*. On the other hand, protein conformation may be a serious bottleneck for heterologous secretion in *L. lactis*. Several publications indicate that conformation change is the major criterion involved in the stabilization of the precursors and the higher yields measured (27–30). It is also possible to display proteins on the bacterial cell wall by several different anchoring methods, each leading to unique host responses as the proteins will be displayed and exposed to gastric degradation differently. The recombinant molecule can be attached to the membrane layer using a transmembrane anchor or a lipoprotein-anchor, or to the cell wall by a covalent link using sortase-mediated anchoring *via* the LPXTG motif (31). The target protein can be synthesized by the *L. lactis*, however, it is also possible to anchor recombinant proteins made in different expression strains by non-covalent binding-domain-mediated anchoring (31–33). This can be extremely useful for delivering proteins that can only be expressed by bacterial strains that are not suited for clinical practice or if PTMs are required that can only be done by eukaryotic cells. The most prevalent PTMs include glycosylation, methionine oxidation, asparagine and glutamine deamidation, and proteolysis. These PTMs not only represent obstacles for precise and reliable bioprocessing but also they may be necessary to induce the appropriate immune responses. The discovery of the *in-trans* surface display system has opened the way to facilitate glycoprotein delivery. This strategy was utilized to produce the tyrosinase related protein-2 (TRP-2-cA) glycoprotein fused with the *L. lactis* N-acetylmuramidase C-terminal LysM cell wall anchor, cA, in mammalian Chinese Hamster Ovary (CHO) cells before subsequent binding to *L. lactis* cell wall (34). *L. lactis*-based secretion of deamidated peptides has also been described. Here, we present the example in which two glutamine residues within the α-gliadin peptide were changed into glutamic acids to stimulate the deamidated immunodominant α-gliadin response for HLA-DQ8 carrying celiac disease patients (35).

Lactic-acid bacteria displaying antigens on their cellular surface have been shown to be able to elicit strong immune responses with one of the most detailed examples being mucosal vaccination against the human papilloma virus (HPV) oncoprotein E7. Mice vaccinated with *L. lactis* expressing the HPV E7 antigen and IL-12 were protected against HPV-16-induced tumors (36). Interestingly, they tested three cellular locations for the oncoprotein expression in *L. lactis* and found that intracellular production ultimately lead to protein degradation and the cell wall-anchored form of E7 gave the strongest immune response (37, 38). A similar therapy, using attenuated *Lactobacillus casei*, was evaluated for safety and efficacy in patients with cervical intraepithelial neoplasia grade 3 (CIN3) as a result of HPV. No adverse events were reported, demonstrating the safety profile of these GM LAB, and oral vaccination induced successful anti-neoplasm mucosal immunity (39). The authors hypothesized that the bacterial cell wall, more specifically the proteoglycan compounds, may display adjuvant properties, thus enhancing the immune response (40). It is possible to increase interactions with host cells by co-expression of adhesive factors on the bacteria cell wall as an adjuvant.

While this way of protein expression opens doors to new approaches, it remains difficult to predict which route is better. An important factor is the amount of protein expressed and how relevant that is when comparing secreted proteins to cell wall-anchored proteins. In the case of bioactive proteins, such as cytokines and hormones, extracellular production will be critical in order to get a functional molecule. Ultimately, it will be necessary to study each protein on a case-by-case basis.

### Probiotic Properties

Probiotic activities (i.e., having health-promoting properties) have been mostly studied in other LAB, such as those of the *Lactobacillus* genus (41). In a recent randomized double-blind placebo trial in human subjects, the probiotic properties of three *Lactobacillus plantarum* strains were studied. All strains influenced the intrinsic repair processes of the small intestinal mucosa on a gene transcriptional level and the strongest effects were demonstrated by *Lactobacillus plantarum* TIFN101 (42). The ability to adhere to the intestinal mucosa is one of the more important selection criteria for probiotics as adhesion is considered to be a necessity for colonization. The *Lactococcus* genus has often been overlooked because it is not part of the normal microflora and it does not proliferate or colonize at a specific location. Nonetheless, there are some studies attributing beneficial anti-inflammatory effects to certain subspecies of *L. lactis* (Figure 1). For example, the *L. lactis* subsp. *lactis* NCDO 2118 reduces symptoms of recurrent colitis in the dextran sulfate sodium (DSS)-induced colitis model. Early IL-6 production may enhance mucosal repair and preserved colonic IL-10 production which could be responsible for the marked anti-inflammatory effects (43). Furthermore, oral treatment with NCDO 2118 nearly doubled the frequencies of CD4^+^CD25^+^LAP^+^ Tregs in mesenteric draining lymph nodes and spleen. Another study found that oral administration of milk fermented with *L. lactis* subsp. *cremoris* FC protected host animals against influenza virus infection (44). The protective effects against influenza virus were mostly derived from the cell components of *L. lactis* subsp. *cremoris* FC and its metabolites, such as exopolysaccharide. Many studies have shown that *L. lactis* can support barrier function in terms of improved mucus, production of antimicrobial peptides, and secretion of soluble immunoglobulin (Ig) A (45).

## *L. Lactis* for Mucosal Delivery of Cytokines and Antigens

One well-studied application of modified *L. lactis* is its potential for treating pathologies of the mucosal layer. IBD refers to the chronic relapsing inflammatory diseases resulting from a breakdown of tolerance in the GIT, and include CD and UC. Many current treatments of IBD approach the pathology by systemic drug administration. Orally administered *L. lactis* can specifically deliver the drug to the lumen of the gut, allowing topical treatment of the inflicted area.

### The Case of the Anti-Inflammatory Cytokine IL-10

One of the first GM *L. lactis* was designed to treat IBD. IL-10 plays a pivotal role in intestinal homeostasis (46) as IL-10^−/−^ mice spontaneously develop colitis (47) and single-nucleotide polymorphisms in IL-10 signaling have been associated with IBD in genome-wide association studies (GWAS) (48–50). Treatment with parenteral administration of recombinant IL-10 showed some efficacy in human trials for CD; however, full remission was never obtained (51–53). Some discrepancies remain concerning the required dose, since high doses exert immunostimulatory effects and lower doses lack clinical efficacy. An important unanswered question regarding systemic IL-10 administration is whether the cytokine reaches its target as the serum half-life of IL-10 is less than 3 h and IL-10 has limited mucosal bioavailability (54). Oral delivery of the recombinant protein might provide an attractive alternative; however, it is complicated by the extreme acid sensitivity of IL-10 in addition to the general complications of oral protein delivery (i.e., degradation in the GIT, hepatic first-pass metabolism and high-production costs). Intragastric administration of IL-10 secreting *L. lactis* (*LL-IL-10)* circumvents many of these issues, bringing the cytokine synthesis directly to the inflamed mucosal tissues. Pioneering work by Steidler et al. showed that with *LL-IL-10* much lower amounts of IL-10 were required to reduce inflammation in DSS-induced chronic colitis, compared with systemic recombinant IL-10. Furthermore, it was possible to prevent disease-onset in IL-10^−/−^ mice (55). One of the many anti-inflammatory effects of IL-10 is the generation of tolerogenic dendritic cells (tDCs), which regulate intestinal homeostasis by inducing suppressor T cells. *LL-IL-10* can modulate immature DCs *in vitro* to upregulate CD83 and CD86, which in turn will induce suppressive helper T cells (Th). This suppressive effect was 40 times more efficient with *LL-IL-10* than exogenous recombinant human IL-10 (56). Even in an *in vitro* setting, where a potentially hostile GI environment is eliminated, it is still more efficient to deliver IL-10 by the *L. lactis* delivery system rather than as a soluble protein.

Based on these results, a small safety and tolerability phase-I clinical trial in 10 CD patients was initiated. Participants were treated with *L. lactis* in which the *thyA* gene was replaced with the human IL-10 gene (24). This presented the first safety test in humans for this biological containment system. Treatment with *LL-IL-10* showed to be harmless, well tolerated and effectively contained *in vivo*, encouraging further use for human application (57). A decrease in disease activity was also observed in 8 out of 10 patients which, combined with a promising safety profile, encouraged further testing. In a larger phase-II trial (NCT00729872) the safety and environmental containment were confirmed, which were the primary objectives of the study. However, no statistically significant differences were observed between placebo and *LL-IL-10* treatment in mucosal healing. The lack of clinical effect may have been due to insufficient exposure to viable bacteria which in turn can be improved by several technological modifications (19, 20, 58).

Perhaps this approach was still not sufficient to protect the synthesized IL-10 from gastric acidity and proteases. A novel tactic for IL-10 delivery by *L. lactis* was designed in 2009, where a plasmid, pValac, was constructed for DNA delivery into eukaryotic host cells. This strategy has demonstrated that expression of the protein encoded in the DNA vector was expressed by epithelial cells of the large intestine (59). Delivery of DNA into the target cells allows for appropriate protein expression (i.e., with the necessary PTMs and correct conformational epitopes) and recognition by the host. The *L. lactis* co-expressing fibronectin binding protein A (FnBPA), which likely acts as an adhesin facilitating plasmid internalization, was slightly more successful and was also able to increase intestinal secretory IgA production, an important first-line of defense mechanism. Nonetheless, this IL-10-encoding DNA plasmid pValac has been cloned into several *L. lactis* strains and was consistently able to diminish intestinal inflammation in a chemically induced murine model for colitis (60).

Another approach to enhance enteric IL-10 production is based on the host immune evasion strategy of the enteropathogenic *Yersinia* species. These bacteria can secrete the soluble protein low-calcium response V antigen (LcrV) that stimulates host-IL-10 production which in turn will facilitate bacterial survival through its anti-inflammatory effects, more specifically on IFN-γ (61). Oral administration of *LL-LcrV* significantly enhanced colonic IL-10 production in a TLR-2-dependent manner and was able to prevent and improve colitis in two different mouse models (62).

Since IL-10 is the key regulator of inflammatory cascades, it has also been studied in other inflammatory diseases outside the GIT. Allergic asthma, the inappropriate immune response to harmless proteins or allergens, is a major health problem with increasing prevalence worldwide (63). It is a hallmark Th2 disorder marked by recruitment of eosinophils, IgE switching, and production of several chemokines that further attract additional leukocytes. Local administration of IL-10 to this respiratory site by means of intranasal administration of *LL-IL-10* was able to modulate acute airway inflammation in a murine model (64). Two xylose-inducible expression systems were tested to target IL-10 to the cytoplasm or extracellular medium (secretion). A decrease of Th2 cytokines (i.e., IL-4, IL-5), a decreased presence of local eosinophils, and improved histology of the lung tissue were observed. Interestingly, these effects were most outspoken with the *L. lactis* strain-producing cytoplasmic IL-10. It is noteworthy that these effects were not mediated by an increase of CD4^+^Foxp3^+^ Tregs. Perhaps increasing *L. lactis* inoculation to more than two exposures could improve therapeutic outcomes and have an effect on Tregs as well.

### TNF-α Antagonists

Systemic treatment with tumor necrosis factor (TNF)-α antagonists, blocking its pro-inflammatory effects, has become a standardized treatment for IBD. However, 10–30% of patients are primary non-responders and 23–46% become secondary non-responders over time, in part due to immunogenicity (65). Furthermore, there are serious side effects leading to a low compliance associated with this therapy (66). Again, the rationale here is that systemic administration requires a much higher dose in order to obtain sufficient amounts at the target site, which is the inflamed GIT. The *L. lactis* has been engineered to secrete mono- and bivalent neutralizing anti-TNF-α nanobodies and is able to ameliorate DSS-induced chronic colitis in mice to a comparable degree as the *LL-IL-10* (67). Interestingly, *LL-anti-mTNF-*α also shows some effects on disease severity in established colitis in IL10^−/−^ mice. The anti-inflammatory effect is limited to the intestine, indicating that there is no generalized immune suppression which would leave patients vulnerable to infections or malignancies.

### Biologic Therapy with IFN-γ

Interferon (IFN)-γ is first and foremost known as a key pro-inflammatory cytokine produced by T cells and natural killer cells. However, it also exerts anti-inflammatory and immunoregulatory activities making it a complex, though interesting, drug target. Sheikh and colleagues demonstrated that IFN-γ exerts its anti-inflammatory properties through inhibition of IL-23. In germ-free mice colonized with enteric microbiota, inhibition of colonic *Il23a* correlated with IFN-γ generation. Colonic CD11b^+^ cells seem to be the main source of IL-23 and are an IFN-γ target (68). Microbial production of soluble and functional recombinant IFN-γ was achieved in *Escherichia coli* (69). While many therapeutic applications require large amounts of recombinant IFN-γ for parenteral use, it has been shown that oral IFN-γ also elicits systemic suppressive effects which cannot be inhibited by circulating antibodies against IFNs (70). The production of mature, biologically active murine IFN-γ by *L. lactis* allows both purification and therapeutic use to target IFN-γ to the mucosal immune system (71). This formulation may prove to be useful in cases where IFN-γ has therapeutic effects on mucosal afflictions, such as oral submucous fibrosis (72).

### Trefoil Factors Taking the Floor

Another successful therapeutic cloned into the *L. lactis* system are the trefoil factors 1, 2, and 3 (TFF1–3), a family of peptides which can promote epithelial wound healing and protect it from further damage, including the mucous epithelia from the GIT. TFFs may be involved in IBD pathogenesis and are a prospective treatment option. Mice treated with *LL-mTFF-1/2/3* after DSS-induced colitis showed reduced neutrophil activation and reduced epithelial damage. Purified TFF was administered rectally and was also able to slightly improve these parameters; however, doses up to 1,200-fold higher than that secreted by *LL-mTFF* were tested and similar efficacy was still not achieved (73). TFFs have also been explored as therapeutics for the treatment of oral mucositis (OM), a painful, debilitating, and common complication associated with cancer treatment with limited treatment options available (74). A phase-II trial that evaluated the efficacy of recombinant human intestinal trefoil factor (rhITF) oral spray for prevention of OM showed the spray was safe, well tolerated, and effective in reducing the frequency and severity of OM in patients with colorectal cancer treated with chemotherapy (75). Human TFF1 was cloned into the *L. lactis* and clinically formulated into a mouth rinse, coded AG013 (76). Encouraging preclinical data from a hamster model formed the basis for a phase-Ib trial (NCT00938080) with AG013 in patients with locally advanced head and neck cancer receiving induction chemotherapy (77). No AG013 bacteria were detected in blood from any subjects, even those given the highest dose (1.2 × 10^12^ CFU/day). In addition, live bacterial load and human TFF1 levels in saliva and oral mucosa were not significantly different between different doses (2.0 × 10^11^; 6.0 × 10^11^; and 1.2 × 10^12^ CFU/day) (77). Preliminary efficacy analysis showed reduced duration of OM, supporting further study in an ongoing phase-II clinical trial (NCT03234465).

An interesting fact about the abovementioned therapies is that much lower doses of therapeutic biologicals are needed when synthesized by a *L. lactis* strain than when the biological exposed to the GIT as a purified protein. The *L. lactis* are able to make close contact with the immune cells of the LP and the intestinal epithelia (IE), which likely means the drug is made available in extremely close proximity to its target cells. Inflammation-induced architectural changes in the mucosa increase this contact, perhaps explaining the widespread success of the *L. lactis* as a mucosal delivery tool for IBD. Oral formulation of cytokine administration, compared with the conventional parenteral route, in its own may be sufficient motivation to give these bugs a closer look.

### Heat-Shock Proteins (HSPs) Revisited

Many treatments with GM *L. lactis* aim to treat the symptoms of excessive inflammation. It is also possible to tackle an underlying pathogenesis of IBD, namely, a breakdown of immune tolerance to microbiota using *L. lactis* (78). In this case, HSPs are extremely interesting antigens to study since they are stress proteins known to be overexpressed in inflamed tissues in IBD (79–81), as well as linkers of pathogen-associated molecular patterns (PAMPs) expressed by commensal bacteria. T-cell-specific inflammatory immune responses to bacterial and human homolog peptides derived from HSP60/65 were found in mucosal biopsies from patients with pediatric CD (82). Circulating IgA antibodies against mycobacterial HSP65 were also elevated in CD patients (83). Clearly, bacterial and/or self-HSP play a part in the pathogenesis of IBD, which motivated their use in a therapeutic setting. Interestingly, HSP boost and direct potent peripherally induced Tregs toward inflammatory sites to reinstate self-tolerance (84). Oral administration of *L. lactis* secreting HSP65 is able to completely prevent DSS-induced colitis in an IL-10/TLR-2-dependent manner (85). Again, CD4^+^LAP^+^ and conventional CD4^+^Foxp3^+^ Tregs were induced.

### Protease Inhibitors

Although many therapies using GM LAB that aim to deliver anti-inflammatory molecules to the intestine showed promising results in murine models for IBD, it is not guaranteed they will also be successful in clinical trials. One technical hurdle might be applicable to all anti-inflammatory cytokines; they must be able to reach the immune cells located in the LP to exert their effects. The DSS-induced colitis model is characterized by severe IE damage. Perhaps in patients, with less severe destruction and exposed mucosal immune system compared with the murine model therapies such as the *LL-IL-10* will not be as efficient. Recent work has shown that high proteolytic activity is found in the intestine of patients with CD and UC. This enhanced proteolytic activity is mostly the result of infiltrating immune cells as well as proteases involved in apoptosis (86). Additionally, there is genetic evidence supporting a role for proteases and protease inhibitors in IBD (87). These observations lead researchers to compare the efficacy of orally administered *L. lactis* secreting the conventional anti-inflammatory cytokines IL-10 and TGF-β with novel strains secreting serine protease inhibitors Elafin and Secretory Leukocyte Protease Inhibitor (SLPI). Interestingly, the GM *L. lactis* secreting Elafin was most successful as it displayed the most significant reduction of inflammation (88). Moreover, endogenous protease inhibitors are also released by the IE, where these therapeutics are delivered, indeed supporting their superior effects compared with IL-10 and TGF-β. Protein quantity, and therefore drug quantity, plays an important role in dose-dependent therapeutics. To address this issue, the authors developed a mutated *L. lactis* strain with an inactivated HtrA protease (htrAΔ). This inactivation led to increased protein production and secretion with only minor effects on bacterial growth. *LL-htrAΔ* secreting Elafin was even more successful in reducing intestinal inflammation, showing that perhaps other therapeutics cloned into a wild-type *L. lactis* might also be more therapeutically effective in a protease-deficient strain.

### SOD and CAT Enzymes

Another contributing factor in the pathogenesis of IBD is oxidative stress signaling which leads to the production of reactive oxygen and nitrogen species that have debilitating effects on the mucosal layer and partake in disease initiation (89). While the human body has natural anti-oxidative capacities, these cannot handle the excessive oxidant load leading to oxidative stress. Some endogenous intracellular antioxidant enzymes, such as superoxide dismutases (SODs), catalase (CAT), and gluthathione peroxidase (GPX), could be used therapeutically to decrease the level of gastrointestinal oxidative stress. In fact, when GM *Lactobacillus casei* modified to produce SOD or CAT were given to mice prior to the induction of trinitrobenzenesulfonic acid (TNBS)-induced CD, they recovered faster and showed lower intestinal inflammation than controls (90). The anti-inflammatory activity of a *Lactobacillus gasseri* strain-producing manganese SOD was shown to be associated with a reduction in the severity of colitis in IL-10-deficient mice. Another ingenious method for SOD delivery was discovered by evaluating the beneficial effects of fermented milk products on murine colitis (91) and on human gut homeostasis (92). Host antimicrobial actions result in lysis of the *L. lactis* subsp. *lactis CNCM I-1631* (*L. lactis* I-1631) bacteria and subsequent release of cytoplasmic SOD that scavenges extracytoplasmic reactive oxygen species and results in colitis attenuation (93).

Considering the abovementioned data, along with others reviewed elsewhere (18, 94, 95), we believe that this clearly underlines the strong potential for *L. lactis* as a tool to deliver active therapeutics to the mucosa to elicit robust local immune effects.

## *L. Lactis* for the Generation and Maintenance of Antigen-Specific Tolerance

A breakdown of antigen-specific tolerance can lead to numerous disorders, including food allergies and autoimmune diseases like MS, arthritis, and T1D. The *L. lactis* carrier showed to be effective in delivering therapeutics to the mucosa, opening the door to study its potential to reinstate systemic antigen-specific tolerance.

### Ovalbumin (OVA) As a Model Antigen

Obtaining antigen-specific tolerance in a therapeutic protocol would be desirable for many diseases which cannot always be prevented. Huibregtse and colleagues showed that it is possible to induce antigen-specific peripheral tolerance by oral administration of *L. lactis* secreting ovalbumin (*LL-OVA)* in OVA-immunized transgenic mice with OVA-specific CD4^+^ T-cell receptors (TCR) (96). Interestingly, *LL-OVA* was able to induce APC-mediated OVA-specific T-cell proliferation at much lower levels than purified OVA. The precise mechanism by which *L lactis* enhances tolerogenic signals remains unclear. However, *LL-OVA* clearly induced a splenic regulatory CD4^+^CD25^−^Foxp3^+^/CTLA-4^+^ population, likely iTregs. This confirms the suggestions that *L. lactis* alters DC functions skewing them toward Treg inducers (56). *L. lactis* may directly modulate antigen processing and presentation as well as the expression of co-stimulatory molecules on DCs. Most of the luminal OVA after *LL-OVA* feeding was found in the cecum and colon, and most of the mucosal OVA was found in the terminal ileum. Currently, it is undetermined which intestinal site is most important for tolerance induction. DCs residing throughout the GIT are able to directly sample luminal antigens through the IE. Due to the association with the IE, *L. lactis* may allow more efficient antigen uptake than is available through oral administration of OVA. Finally, antigen-specific IL-10 production was only observed in mice treated with *LL-OVA* (compared with an empty vector control or purified antigen) and OVA-specific suppression was dependent on TGF-β, a hallmark characteristic of Th3 cells. Again the *L. lactis* empty vector control itself had some effects, being able to significantly reduce the delayed-type hypersensitivity response to OVA as well as moderately decrease OVA-specific CD4^+^ T-cell proliferation. Collectively, these findings highlight how different Tregs (iTregs and Th3) can overlap in functionality and phenotype and hint at the complexity of the regulatory pathways involved in mucosal (oral) tolerance. Moreover, these promising results were obtained in a TCR-transgenic mouse and are not guaranteed to be replicable in a host with a normal, broad TCR repertoire. Nonetheless, if this tool can induce Tregs that can confer bystander suppression to T cells reactive to unknown or multiple antigens, this approach would be extremely valuable (97).

### Food and Inhaled Antigens

Several immune-mediated diseases (such as type-I allergies) are triggered by well-defined antigens. Therefore, tolerance protocols aiming at targeting these food- or inhaled airborne antigens are clearly warranted. It was shown that early feeding or intranasal administration of high antigen doses could induce tolerance in mice and that tolerized mice had more IL-10- and TGF-β-producing T cells in their Peyer patches (1, 98). A valuable asset of *L. lactis* as bacterial delivery vehicles for vaccines is their potential to elicit antigen-specific secretory IgA responses at mucosal surfaces. Intranasal or oral inoculation of mice with *L. lactis* engineered to produce β-lactoglobulin (*LL-BLG*), a major allergen in cow’s milk, induced specific anti-BLG fecal IgA antibodies (28). Furthermore, pretreatment with *LL-BLG* in the presence of IL-12-producing *L. lactis (LL-IL-12*) prevented a Th2-type immune response after systemic sensitization with BLG by developing a strong Th1 response that correlated with the amount of recombinant BLG produced (99, 100). Delivery of a deamidated gliadin epitope, an immunodominant epitope in celiac disease, by *L. lactis* to transgenic humanized non-obese diabetic (NOD) AB°DQ8 mice was also able to induce antigen-specific tolerance mediated by Foxp3^+^ Tregs that function in an IL-10 and TGF-β-dependent mechanism (35). While some probiotic LAB, *Lactobacillus reuteri* and *Lactobacillus casei*, can prime DCs to drive the development of IL-10-producing Tregs, supplying IL-10 to the intestine by delivery using *L. lactis* is also an option (101). Indeed, oral administration of *LL-IL-10* diminished anaphylaxis significantly in an animal model of food allergy. Preventative treatment with *LL-IL-10* inhibits antigen-specific serum IgE and IgG_1_ production and increases antigen-specific GI IgA levels (102). Interestingly, some of the immune effects can be attributed to the *L. lactis* since the wild-type control also reduces antigen-specific antibodies and moderately increases IL-10 secreting cells in the Peyer’s patches.

With respect to inhaled allergens, the modulation of allergic immune responses to the major dust mite allergen Der p2 by recombinant *L. lactis* has recently been described (103).

### Autoantigens: *L. lactis* As a Potential Immunotherapy for Autoimmune Type 1 Diabetes

Type 1 diabetes is a chronic autoimmune disease characterized by immune-mediated destruction of the pancreatic insulin-producing beta cells by autoreactive CD4^+^ and CD8^+^ T cells (104). The eventual total loss of insulin production causes patients to become reliable on exogenous insulin to manage their glycemia levels. The prevalence of T1D is estimated to be 20 million patients worldwide, with an alarming increase in incidence rate in children younger than 5 years old (105). Treatment with exogenous insulin is successful in bringing the glycemia to normal levels, both in fasting and postprandial settings. However, vascular complications, both of the macrovascular and microvascular blood vessels, are responsible for the morbidity and mortality of T1D (106). These life-threatening complications and rise in incidence emphasize the need for a cure.

#### Therapies Broken Down by Disease Stage

Novel immunotherapies aim to restore antigen-specific tolerance without notable immune suppression. Which therapeutic approach is taken depends heavily on the disease stage which correlates with the rate of beta cell decline (107). The aim of *primary prevention* is to prevent islet autoimmunity in genetically susceptible young individuals. *Secondary prevention* protocols aim to prevent autoantibody-positive individuals from progressing to overt dysglycemia. In *tertiary intervention* protocols, the goal is to minimalize further beta cell loss and improve glycemic control after diagnosis (107). Carrying out trials at these different stages each come with their own limitations, such as extensive screening to identify the target population for primary and secondary preventions and limited therapeutic benefit in the case of success for tertiary preventions. Since autoimmunity, marked by the presence of autoantibodies produced by B cells, is present before onset of clinical symptoms this is an appropriate stage of the disease for antigen-specific immune interventions (108). This does not exclude their use in a tertiary intervention stage when combined with islet supplementation to ensure sufficient beta cell mass is present to reach normal glycemic control in the case of successful immune modulation.

Nowadays, the use of anti-CD3 monoclonal antibodies (mAbs) has moved from the bench to the bedside. Initial studies with anti-CD3 mAbs demonstrated that a short-term treatment with a low dose (5 μg/day intravenously for five consecutive days) could induce durable tolerance to beta cell antigens without inducing general immunosuppression in preclinical models (109). Anti-CD3 mAbs did not remove the pancreatic insulitic lesions but were ineffective in prophylactic settings, indicating that the timing of treatment with anti-CD3 is critical for inducing long-term tolerance. Therefore, researchers are currently evaluating the window of opportunity for anti-CD3 therapy, with a phase-II trial (NCT01030861) to evaluate subjects further from the time of initial diagnosis. The first anti-CD3 mAb, OKT3, was used to reduce graft rejection after transplantation. Due to its Fc receptor (FcR) binding properties, it induced a cytokine storm making it unsuitable for clinical use. Two humanized non-mitogenic anti-CD3 mAbs, teplizumab and otelixizumab, were developed for clinical trial testing. Phase-III trials in new-onset T1D patients showed a degree of clinical efficacy demonstrated as better C-peptide response, lower insulin requirements and better glycemic control. However, these mAbs failed to meet their primary endpoints, such as significant change in clinical outcome (110–113). Based on these observations, it seems that these agents alone do not restore normal glucose control, and future approaches will likely require combinations of agents with complementary immune or metabolic activity.

#### Targeted versus Ignored Beta Cell Antigens

An important determinant for the success of an antigen-based immune intervention is the choice of antigen. Antigen-specific therapies have mainly concentrated on administering the autoantigens themselves. The most common and abundant autoantigens in T1D patients and high-risk individuals are (pro)-insulin (P)INS, glutamic-acid decarboxylase of 65 kDa (GAD65), tyrosine phosphatase-like protein ICA152 (IA-2), and zinc transporter 8 (ZnT8) (114, 115). These epitopes are the target of autoantibodies and can activate autoreactive CD4^+^ and CD8^+^ T cells. Already more than 25 years ago it was shown that oral administration of insulin was effective in delaying the onset and decreasing the incidence of diabetes in NOD mice (116). Since then many variations of insulin administration, as well as several other autoantigens, were tested in animal models with overall very positive results (117). These encouraging results led to numerous clinical trials that all failed to meet their primary endpoints [reviewed in Ref. (107)]. It became clear that successful clinical translation of antigen-specific therapies would rely on a variety of factors, such as antigen selection, antigen dose, antigen bioavailability, route of administration, and timing of intervention (118). Moreover, since the ability of beta cell autoantigens to prime the immune system diminishes with disease development, beta cell antigens that are not uninvolved in the autoimmune process can avoid disease more successfully in NOD mice. Oral delivery of T1D-relevant antigens *via* the *L. lactis*, as a means to circumvent these pharmacokinetic limitations, together with HSP65 was already proven successful in reducing diabetes incidence in NOD mice in an antigen-dependent manner (119, 120).

Over the years, it has become clear that the immunological defects of T1D are complex and to halt or prevent T1D in humans in which T1D pathogenesis appears to be very heterogeneous will require more than one single agent. The T1D community advocates the use of combination immunotherapies targeting multiple biological pathways in a synergistic manner (121). We propose that mucosal administration of T1D-relevant autoantigens in combination with low doses of systemic immune modulators and/or anti-inflammatory agents would be a means to restore long-term antigen-specific tolerance while minimizing the risk of side effects (108, 122).

#### Mechanism of Action? Biomarkers of Success?

Our group demonstrated that oral administration of *L. lactis* secreting PINS and IL-10 (2 × 10^9^ CFU/day/6 weeks) combined with systemic low-dose anti-CD3 (2.5 μg/day/5days) (combi-PINS therapy), stably reversed new-onset diabetes in around 60% of NOD mice (123, 124). Compared with anti-CD3 monotherapy, combi-PINS reverted diabetes faster and cured mice had more stable glycemia levels during therapy and the follow-up period. This was also shown for oral delivery of *LL-GAD65_370–575_* + *IL-10* with anti-CD3 (combi-GAD65 therapy) in a similar manner (125). Both combination therapies were well tolerated showing no signs of weight loss or intestinal inflammation. A remarkable observation is that combi-GAD65_370–575_ had a higher efficacy in mice with severe hyperglycemia at diagnosis (>350 mg/dl) than anti-CD3 alone as well as combi-PINS, alluding to the importance of antigen choice. Our data imply that splitting a large autoantigen may expose several cryptic elements and prime more efficiently regulatory responses than the whole autoantigen. Inducing regulation to specific beta cell autoantigenic epitopes may be safe as (1) the regulatory immune responses will be specific and (2) it is less likely to boost autoreactive T-cell responses since cognate T cells are not previously activated.

This therapeutic effect was not accompanied with proliferation of functional beta cells but rather a preservation of beta cell mass and a reduction in severe insulitis. Coadministration of gut-delivered IL-10 *via L. lactis* also improved reversal rates. The importance of gut-specific IL-10 in the balance between the intestinal mucosa and the immune system was demonstrated by the development of transgenic mice (Fabpi-IL-10 mice) that overexpress this cytokine only in their IE (126). Compared with wild-type mice, Fabpi-IL-10 mice had high numbers of intraepithelial lymphocytes (IELs) and IgA-producing B cells in their LP. Activated IELs in Fabpi-IL-10 mice had lower levels of Th1 cytokines TNF-α and IFN-γ but increased levels of the Th2 cytokine TGF-β. These data provide evidence for an *in vivo* lympho-epithelial cross-talk, by which cytokines locally produced by intestinal epithelial cells (IECs) can regulate intestinal immune responses without systemic modifications. Certainly, intestinal Tregs are targeted by the mucosal delivery of IL-10 and it may also directly modulate Th17 cells since these cells also express IL-10 receptors (127).

**Figure 2:**
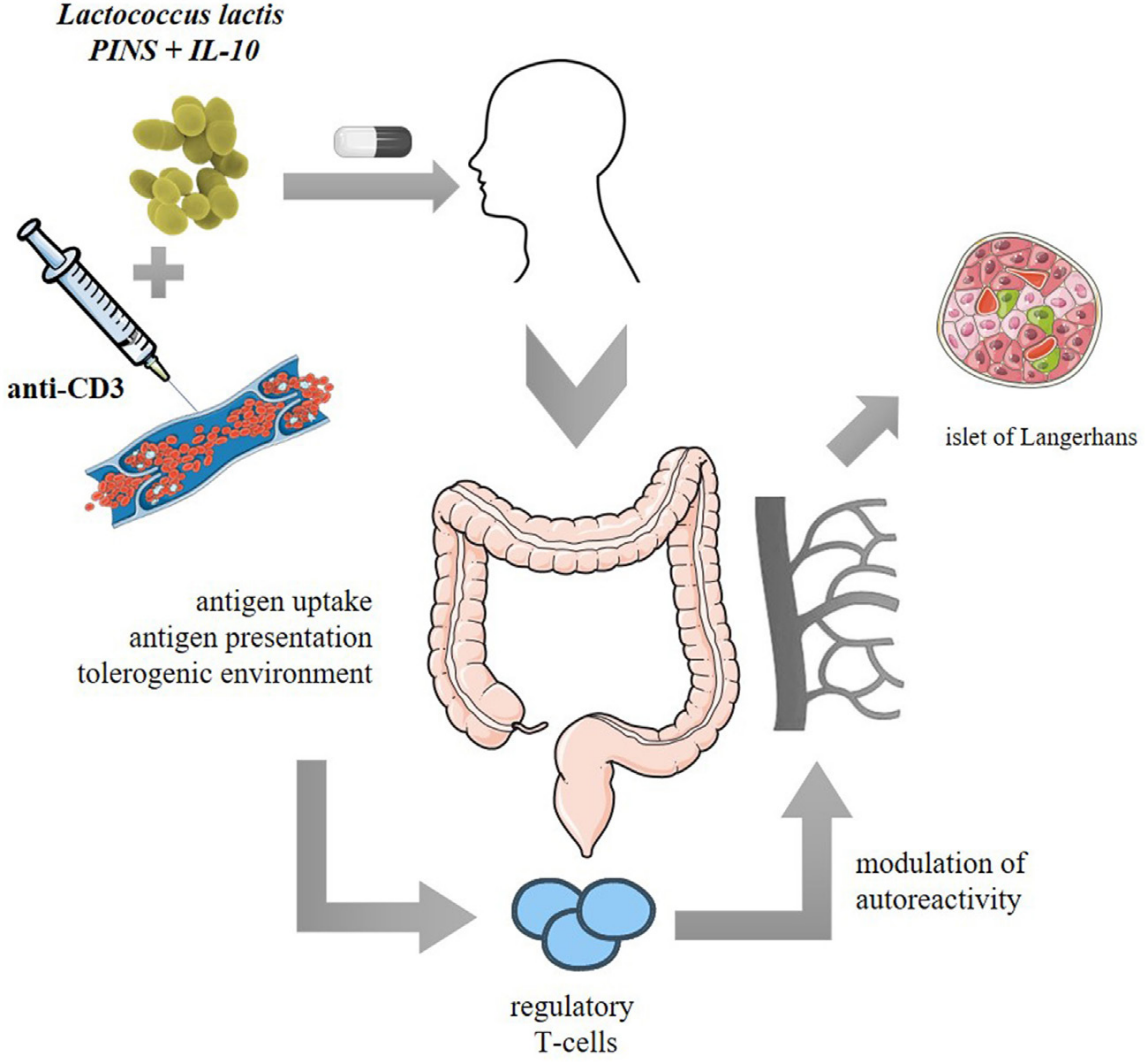
Proposed clinical model of *Lactococcus lactis*-based vaccine or immunotherapy: oral administration of freeze-dried encapsulated biologically contained *L. lactis* modified to secrete human proinsulin (PINS) and interleukin-10 (IL-10) combined with intravenous systemic monoclonal CD3 antibodies (anti-CD3). PINS together with IL-10 may be presented to migratory CD103^+^ dendritic cells which will home to the mesenteric lymph nodes and mediate priming of Foxp3^+^ regulatory T cells (Tregs). The induced Tregs will proliferate and acquire tolerogenic properties when migrating back to the lamina propria and will thereafter be released in the circulation to modulate islet beta cell autoreactivity in a Foxp3^-^ and IL-10-dependent manner.

T-cell responsiveness to disease-unrelated antigens was not altered as unmanipulated NOD mice and combi-PINS-cured mice displayed similar responses *in vitro* to alloantigen stimulation and were equally able to reject allogeneic skin transplants (123). Moreover, no deletion or anergy of autoreactive effectors was observed after combi-GAD therapy as adoptive transfer of CD25-depleted splenocytes from cured combi-GAD65_370–575_-treated mice induced diabetes in NOD/SCID recipients (125). An earlier study showed that intranasal PINS administration when combined with systemic anti-CD3 successfully induced long-term reversion of diabetes in around 50% of both RIP-LCMV and NOD mice and this reversal was linked to induction of PINS-responsive Tregs (128). Indeed, treatment with *L. lactis*-based combi-PINS increases antigen-specific functional CD4^+^CD25^+^Foxp3^+^ Tregs which homed to the pancreas (Figure 2).

Furthermore, two predictive biomarkers were discovered for therapeutic success, namely, glycemia values at onset which reflects residual beta cell mass and insulin autoantibody (IAA) positivity (124). *Post-hoc* analysis of the Diabetes Prevention Trial-Type 1 (DPT-1), where at risk children were fed high doses of insulin, also indicated that it may be necessary to select individuals with high IAA levels for antigen-specific trials with insulin (129). This consensus should probably be generalized to identifying autoantigen reactivity in patients to select appropriate antigen-specific therapies. Finally, the clinical-grade combi-PINS therapy induces functional IL-10-secreting Foxp3^+^ (CD25^+^ and CD25^-^) Tregs in the pancreatic draining lymph nodes and the pancreas, irrespective of metabolic outcome. On the other hand, CTLA-4^+^ frequency among Treg subsets was only increased in mice responsive of therapy. Foxp3^+^ Treg frequencies were increased in the periphery of treated mice compared with newly diabetic controls, especially in therapy responders, alluding to the potential of this population as an immune biomarker for therapy. Additionally, Foxp3^+^ T cells were shown to be crucial for both induction and maintenance of *L. lactis*-based combi-PINS tolerance.

We believe the in-depth characterization of mechanisms of action of the safe *L. lactis-*based combination therapy combined with the abovementioned biomarkers for predicting therapeutic success validate this therapy as a suitable intervention for T1D that is ready for clinical testing.

## Translation to Clinic

The preclinical efficacy data obtained from animal models are very encouraging, but it is important not to forget the significant differences with humans. Changes in dose, composition, and administration form can have substantial effects on drug potency. It is also important to keep in mind the type of animal and disease model that was used. Data from humanized mice will provide a better prediction of effects expected to be seen in human trials. On the other hand, chemically induced disease models may also not mimic the entire pathology, like DSS-induced colitis which can even happen in the absence of adaptive immunity.

Both *L. lactis*-based combination therapies, combi-PINS and combi-GAD65, hold tremendous promise as they effectively reverse T1D in an auto-antigen-specific manner without inducing general immune suppression. However, successful clinical translation requires the generation of a *L. lactis* strain suitable for patient use and the identification of certified biomarkers for both immunological and therapeutic success. Recently our group tested such a self-containing clinical-grade *L. lactis* strain, meaning chromosomal integration of human PINS and IL-10 sequences (24), in an intervention protocol with low-dose anti-CD3 in NOD mice (124). The safety profile of GM *L. lactis* strains will be of utmost importance if they are to be used to treat new-onset T1D patients, which are often young children. *L. lactis* bacteria are, as is discussed in detail above, harmless of nature and have been ingested even at high doses by healthy children, adults, elderly, as well as immune-compromised individuals and showed no health compromising issues (Figure 1). To date several clinical trials using live GM *L. lactis* have been completed showing clearly that treating patients with mucosal pathologies was safe and well tolerated (Table 1). These trials demonstrated that the *thyA-*containment system designed in 2000 was effective at restricting environmental dissemination and pharmacokinetic assessment confirmed an adequate formulation for human use was found. The pharmacokinetic profile of *LL-IL10* was also tested in healthy and colitic mice and even in cases of severe intestinal inflammation (and therefore gut leakiness) no *L. lactis* were found in the circulation (130). Toxicity studies performed in mice and primates showed no evidence for anti-hIL10 antibodies and the no observed adverse effects level (NOAEL) was the highest dose given (130). Of note, *L. lactis* are also being tested in a phase-I trial (NCT02958540) as non-live non-GM carriers to deliver antigens to the nasal mucosa as a vaccination strategy against respiratory syncytial virus (131) (Table 1).

**Table 1:** Clinical studies using *Lactococcus lactis* as carriers to target mucosal delivery of heterologous proteins.

	Strain	Heterologous protein secretion	Expression system	Inflammatory condition	Administration	Clinical trial	Outcome	Reference	Clinical trial identifier
Live genetically modified *L. lactis*	*L. lactis* Thy 12	hIL-10	ThyA native promoter from *L. lactis*	Crohn’s disease	Oral capsule	Phase-I trial evaluating safety and biologic containment of the transgene in patients with Crohn’s disease	Treatment was safe and well tolerated, furthermore bacterial growth after passage through the GI tract was dependent on thymidine indicating the environmental containment system is effective	Preclinical data (55) Clinical data (57)	
	*L. lactis* AG011	hIL-10	ThyA native promoter from *L. lactis*	Ulcerative colitis	Oral capsule	Phase-IIa trial to evaluate the safety tolerability, pharmacodynamics, and efficacy of AG011 in patients with ulcerative colitis	Primary endpoints were met, confirming safety and environmental containment. However, no statistical significant effects on mucosal healing were observed	ActoGenix press release (October 9th 2009)	NCT00729872
	*L. lactis* AG013	hTFF1	ThyA native promoter from *L. lactis*	Oral mucositis	Oral rinse	Phase-I trial in healthy volunteers to evaluate the pharmacokinetic profile of orally delivered AG013	The PK profile showed live AG013 bacteria adhere to the oral mucosa and actively secrete protein for up to 24 h. Food intake reduced exposure while intake of a beverage lid not	ActoGcni_X_ press release (August 22nd 2012)	
	*L. lactis* AG013	hTFF1	ThyA native promoter from *L. lactis*	Oral mucositis	Oral rinse	Phase-Ib trial to assess safety and tolerability of topically applied AG013 in oral mucositis in subjects receiving induction chemotherapy for the treatment of cancers of the head and neck	Treatment was safe, as no AG013 bacteria were detected in blood. Compliance was in accordance with daily dosing frequency and preliminary efficacy data were reported	Preclinical data (76) Clinical data (77)	NCT00938080
	*L. lactis* AG013	hTFF1	ThyA native promoter from *L. lactis*	Oral mucositis	Oral rinse	Phase-II trial to determine efficacy, safety and tolerability of AG013 in oral mucositis compared with placebo when administered three times per day	Actively recruiting, estimated primary completion date May 2020		NCT03234465
	*L. lactis* AG014	Anti-TNF-alpha (Certolizumab)	Not disclosed	Inflammatory bowel disease	Oral capsule	Phase-I trial studying safety and tolerability, medical endoscopic sampling methodology and characterization of the pharmacokinetic profile of oral doses of AG014 in healthy volunteers	Showed high safety and tolerance levels while also showing live AG014 were targeted to the GI tract and localized exposure of anti-TNF-alpha was efficiently measurable by endoscopic sampling	Preclinical data (67) ActoGeni_X_ press release (October 15th 2014)	
Non-live non-genetically modified *L. lactis*	Bacterium-like particles derived from inactivated *L. lactis*	RSV fusion protein F	Protein domain fused to heterologous protein will bind non-covalently to lactococcal peptidoglycan	Respiratory syncytial virus	Intranasal spray	Phase-I trial to assess the safety. reactogenicity and tolerability of two intranasal dose levels of SynGEM^®^ in healthy volunteers	Estimated primary completion date December 2017	Preclinical data (131) Mucosis press release (November 7th 2016)	NCT02958540

*GI, gastrointestinal; hIL-10, human interleukin-10; hTFF1, human trefoil factor 1; L. lactis, Lactococcus lactis; PK, pharmacokinetic; RSV, respiratory syncytial virus; TNF, tumor necrosis factor*.

Clinical trial feasibility will rely on bringing preclinically tested laboratory strain of *L. lactis* from the bench to bedside. One hurdle to overcome is stable storage of large amounts of temperature-sensitive *L. lactis*. Freeze-drying, or lyophilization, reduces water activity in bacteria and allows long-term and low-cost storage at temperatures above freezing (132). This formulation also improves passage through the GIT; however, it can also significantly reduce viability. On the other hand, a different modification, namely, the accumulation of intracellular trehalose to improve bile resistance, concurrently improved viability under this formulation (20). Such technical adaptations are the cornerstone of successful therapeutic clinical translation.

The obtained safety data are reassuring in moving forward with clinical testing of live GM *L. lactis* bacteria. Furthermore, significant progress has already been made to develop optimal formulations suitable for human use.

## Conclusion

*Lactococcus lactis* have evolved from agents used in the food industry into qualified vehicles for mucosal drug delivery. However, several technological advancements were necessary for this transition, such as the identification of a strong constitutive promoter as well as several inducible expression systems. Furthermore, heterologous protein expression was shown to be possible at a handful of cellular locations. These modifications put the *L. lactis* on the map as an extremely versatile vehicle. Nonetheless, the industrial convenience is only transcended by the safety profile these bacteria have demonstrated in clinical trials. A proven effective environmental containment system, through replacement of the lactococcal *thyA* gene, is reassuring with respect to concerns regarding clinical use of GM organisms. Therefore, it is possible to carefully move forward with the substantial successful preclinical results obtained using GM *L. lactis* as immunotherapeutic tools.

## Author Contributions

DC and CG drafted the manuscript and CM critically revised the manuscript.

## Conflict of Interest Statement

The authors declare that the research was conducted in the absence of any commercial or financial relationships that could be construed as a potential conflict of interest.

## Abbreviations

APC, antigen-presenting cell; CAT, catalase; CD, Crohn’s disease; CIN3, cervical intraepithelial neoplasia grade 3; DC, dendritic cell; DSS, dextran sulfate sodium; FcR, Fc receptor; FDA, Food and Drug Administration; GAD65, glutamic-acid decarboxylase of 65 kDa; GALT, gut-associated lymphoid tissue; GIT, gastrointestinal tract; GM, genetically modified; GPX, glutathione peroxidase; GRAS, generally regarded as safe; GWAS, genome-wide association studies; HPV, human papilloma virus; HSP, heat-shock protein; IA-2, tyrosine phosphatase-like protein ICA152; IAA, insulin autoantibody; IBD, inflammatory bowel disease; IE, intestinal epithelia; IEC, intestinal epithelial cell; IEL, intraepithelial lymphocyte; IFN, interferon; Ig, immunoglobulin; IL, interleukin; iTreg, induced regulatory T cell; *L. lactis*/LL, *Lactococcus lactis*; LAB, lactic-acid bacteria; LP, lamina propria; mAb, monoclonal antibody; MS, multiple sclerosis; NICE, Nisin-controlled gene expression; NOD, non-obese diabetic; OM, oral mucositis; OVA, ovalbumin; PAMP, pathogen-associated molecular patterns; PINS, proinsulin; PTM, posttranslational modification; SOD, superoxide dismutase; SP, signal peptide; T1D, type 1 diabetes; TCR, T-cell receptor; tDC, tolerogenic dendritic cell; TFF, trefoil factors; TGF, transforming growth factor; Th, helper T cell; TLR, toll-like receptor; TNBS, trinitrobenzenesulfonic acid; TNF, tumor necrosis factor; Treg, regulatory T cell; UC, ulcerative colitis; ZnT8, zinc transporter 8.
